# Comparing the Effectiveness and Safety of Remdesivir and Molnupiravir in COVID‐19: A Systematic Review and Meta‐Analysis

**DOI:** 10.1002/iid3.70289

**Published:** 2025-10-16

**Authors:** Seyed Hamid Pakzad Moghadam, Ali Sarkoohi, Zia Navidi, Bahman Amani, Behnam Amani, Saeed Khorramnia

**Affiliations:** ^1^ Department of Anesthesiology, School of Medicine Rafsanjan University of Medical Sciences Rafsanjan Kerman Iran; ^2^ Department of Health Management and Economics, School of Public Health Tehran University of Medical Sciences Tehran Tehran Iran

**Keywords:** COVID‐19, effectiveness, molnupiravir, remdesivir, safety, SARS‐CoV‐2

## Abstract

**Background:**

Remdesivir and molnupiravir have been approved and are being used as viable treatment options for patients with coronavirus disease 2019 (COVID‐19). This systematic review and meta‐analysis sought to evaluate and compare the safety and effectiveness of these two antiviral drugs in the treatment of COVID‐19.

**Methods:**

An extensive search was conducted across several databases, including Web of Science, PubMed, the Cochrane Library, and medRxiv, up to July 1, 2024. To evaluate the risk of bias, a standardized bias assessment tool was used. Data from the selected studies were analyzed using Comprehensive Meta‐Analysis software.

**Results:**

The analysis included data from 10 studies, encompassing a total of 5766 patients. According to the meta‐analysis, remdesivir and molnupiravir showed no statistically significant differences in mortality (odds ratio [OR] = 2.54, 95% confidence interval [CI]: 0.67, 9.57), hospitalizations (OR = 2.43, 95% CI: 0.81, 7.24), viral clearance rates (OR = 1.49, 95% CI: 0.64, 3.46), or average time to viral clearance (standardized mean difference = 0.02, 95% CI: −0.40, 0.46). However, the incidence of adverse events was lower in the remdesivir group (OR = 0.49, 95% CI: 0.26, 0.93). The certainty of evidence for these findings was evaluated and determined to be low or moderate.

**Conclusion:**

The findings of this study suggest that remdesivir and molnupiravir have similar effectiveness in treating COVID‐19 outpatients; however, molnupiravir is associated with a higher rate of adverse events. Additional studies are required to enable a more thorough evaluation of these treatments for COVID‐19.

## Graphical abstract

A systematic review and meta‐analysis evaluating the effectiveness and safety of Remdesivir and Molnupiravir as treatments for COVID‐19 found no significant differences between the two drugs in mortality, hospitalization, viral clearance rates, or mean viral clearance time. However, the study indicated that further research is needed to confirm or challenge these findings.

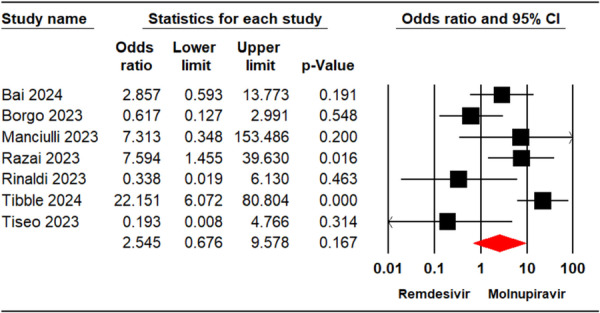

## 1 Introduction

Although the widespread implementation of vaccinations against the severe acute respiratory syndrome coronavirus 2 (SARS‐CoV‐2) has significantly reduced the global mortality and morbidity rates associated with coronavirus disease 2019 (COVID‐19), certain populations remain vulnerable to SARS‐CoV‐2 infection due to insufficient protection [1, 2]. Therefore, it is imperative to carefully consider the integration of effective therapeutic interventions alongside SARS‐CoV‐2 vaccines for these at‐risk groups [3]. Several therapeutic resources have been explored for managing COVID‐19, including antiviral agents, as well as other drug classes that may mitigate disease severity. These include macrolides, such as azithromycin, which have shown potential benefits in reducing inflammation [4], anti‐inflammatory drugs, both steroidal (e.g., dexamethasone) and nonsteroidal [5], and biologics like tocilizumab [6], alongside other viral drugs [7]. These therapies have been investigated for their ability to lessen the severity of COVID‐19, complementing the primary antiviral treatments. Several antiviral agents have been proposed as viable treatment options for managing COVID‐19 patients [8, 9, 10, 11]. Molnupiravir, an orally administered antiviral medication, is specifically indicated for treating adult patients with mild‐to‐moderate COVID‐19 who are at a heightened risk of progressing to severe illness and are unable to access or utilize other approved treatments for COVID‐19 [12]. In contrast, remdesivir, an approved antiviral drug, is authorized for u treating COVID‐19 in both adolescents and adults [13]. The World Health Organization (WHO) recommends molnupiravir for non‐severe COVID‐19 in adults at high risk for hospitalization with symptoms for less than 5 days when alternative treatments are unavailable, while suggesting remdesivir may reduce hospitalization rates more effectively in high‐risk patients [14]. The National Institutes of Health endorses molnupiravir (800 mg orally twice daily for 5 days) as an alternative for nonhospitalized adults with mild‐to‐moderate COVID‐19 at high risk when nirmatrelvir/ritonavir or remdesivir are unsuitable, starting within 5 days of symptom onset; remdesivir is recommended for nonhospitalized patients within 7 days of symptom onset for 3 days and for hospitalized patients for 5–10 days based on clinical response [15]. The Infectious Diseases Society of America suggests molnupiravir for ambulatory adults with mild‐to‐moderate COVID‐19 at high risk when other options (e.g., nirmatrelvir/ritonavir or remdesivir) are unavailable, starting within 5 days, and recommends remdesivir for both ambulatory and hospitalized patients within 7 days of symptom onset. Both drugs are primarily indicated for high‐risk outpatients with mild‐to‐moderate COVID‐19, with remdesivir also used in hospitalized settings and molnupiravir as a secondary option [16]. Numerous studies have substantiated the effectiveness of both molnupiravir and remdesivir in enhancing clinical outcomes among COVID‐19 patients [17, 18, 19, 20]. However, Real‐world investigations comparing the effectiveness of remdesivir and molnupiravir have yielded conflicting results [21, 22, 23, 24]. For example, studies by Borgo et al. [21] and Tiseo et al. [24] suggest that remdesivir is associated with superior outcomes, while research by Manciulli et al. [22] indicates that molnupiravir may be more effective. Given these inconsistencies, a systematic review and meta‐analysis are needed to determine which intervention, remdesivir or molnupiravir, is more effective in treating COVID‐19 patients. This study aims to compare the safety and effectiveness of these two treatments for patients with COVID‐19.

## 2 Methods

The current research adhered to the Preferred Reporting Items for Systematic Reviews and Meta‐Analyses (PRISMA) statement as the basis for the systematic review and meta‐analysis [25]. The study protocol was registered on PROSPERO with the registration number CRD42024569366.

### 2.1 Literature Search

A comprehensive search for relevant evidence up to July 1, 2024, was conducted independently by two researchers, following a systematic approach to ensure thoroughness and accuracy. The search involved specific keywords across multiple databases, including Web of Science, PubMed, and the Cochrane Library, which are recognized for their extensive collections of peer‐reviewed literature. In addition to these primary sources, further records were identified by searching medRxiv and Google Scholar, which provide access to preprints and a broader range of research articles. To ensure a comprehensive search, references from existing systematic reviews and key studies were checked for additional relevant records. The keywords employed in this extensive literature search encompassed “SARS‐CoV‐2,” “COVID‐19,” “Remdesivir,” and “Molnupiravir,” with restrictions applied to include only English‐language articles. The search strategies tailored to specific resources are documented in the Supplementary file.

### 2.2 Study Selection

The systematic review and meta‐analysis encompassed studies that fulfilled the following criteria: patients who had a confirmed positive polymerase chain reaction (PCR) test for COVID‐19, received either remdesivir or molnupiravir as a standalone treatment, and reported relevant safety and effectiveness outcomes, including mortality rates, hospitalization rates, and adverse events. Any discrepancies between the researchers regarding study eligibility were resolved through discussion, and if necessary, a third reviewer was consulted to ensure that the selection process was fair and unbiased. Studies were excluded if they reported irrelevant outcomes, involved combination therapies, were case reports, or included healthy individuals.

### 2.3 Risk of Bias Assessment and Quality of Evidence

The potential bias in the studies included in the analysis was independently evaluated by two researchers utilizing the Risk of Bias in Nonrandomized Studies of Interventions (ROBINS‐I) tool [26]. This comprehensive tool assesses several domains, including confounding, selection bias, measurement bias, and reporting bias, enabling a thorough evaluation of the methodological quality of the studies. Each domain was carefully examined to identify potential sources of bias that might compromise the validity of the results. Furthermore, the strength of evidence for each outcome was evaluated using the Grading of Recommendations, Assessment, Development, and Evaluations (GRADE) tool. This widely recognized framework provides a systematic approach to grading the quality of evidence and making recommendations based on the strength of the evidence. The GRADE tool categorizes evidence into four levels: very low, low, moderate, and high quality, based on criteria including study design, risk of bias, inconsistency in findings, indirectness, and imprecision [27]. The assessment process involved detailed discussions between the researchers to reach consensus on any discrepancies in their evaluations. This collaborative approach aimed to enhance the reliability of the bias assessments and ensure that all relevant factors were considered. By employing both the ROBINS‐I and GRADE tools, the study aimed to provide a robust evaluation of the quality of the included studies and the reliability of the outcomes reported.

### 2.4 Data Extraction

Data extraction was independently conducted by two researchers using a standardized form designed to capture all relevant information, including study characteristics, patient details, treatment interventions, effectiveness outcomes, and safety outcomes. The information extracted encompassed essential details such as study location, patient demographics (age, gender, underlying health conditions, disease severity at baseline), treatment details (dosages, duration of therapy, concomitant medications), effectiveness outcomes (mortality rates, hospitalization rates, time to clinical improvement), and safety outcomes (incidence and severity of adverse events).

### 2.5 Data Analysis

Data analysis was conducted using the Comprehensive Meta‐Analysis software (version 3.0) to compare the safety and effectiveness of remdesivir versus molnupiravir in COVID‐19 patients. In the analysis of dichotomous variables, the odds ratio (OR) accompanied by a 95% confidence interval (CI) was applied to facilitate the comparison of event rates across the two treatment groups. For continuous data, the standardized mean difference (SMD) with a 95% CI was utilized, enabling the assessment of differences in means across studies while accounting for variability in measurement scales. To assess the degree of heterogeneity among the studies, the *I*² statistic was calculated, with high heterogeneity defined as *I*² > 50% or a *p*‐value < 0.1. When high heterogeneity was detected, a random‐effects model was applied to account for variability between studies, allowing for more generalized conclusions. Conversely, when studies exhibited low heterogeneity, the fixed effects model was utilized, providing a more precise estimate of the treatment effects under the assumption that all studies are estimating the same underlying effect. Furthermore, sensitivity analyses were conducted individually for each outcome to evaluate the reliability of the results. This involved systematically excluding each study from the analysis to determine how its absence would impact the overall results.

## 3 Results

### 3.1 Search Result

**Figure 1:**
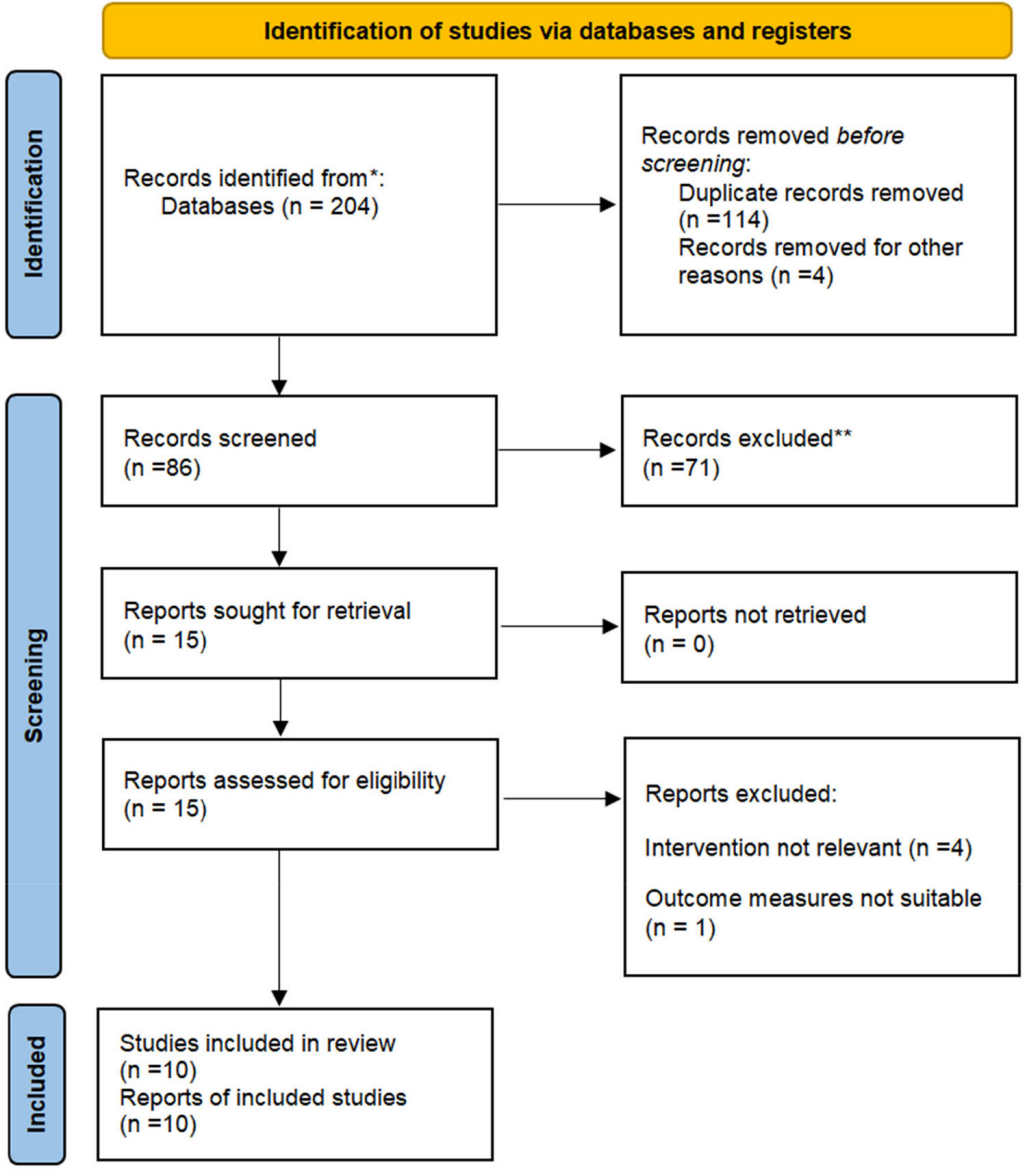
PRISMA flow diagram.

The search results, illustrated in Figure 1, outline the systematic process of selecting studies based on their titles, abstracts, and full‐text reviews. Initially, duplicate records were removed, resulting in 86 articles being screened against the inclusion criteria. Of these, 15 studies qualified for full‐text review; however, 5 were subsequently excluded according to the criteria specified in Figure 1. Ultimately, 10 studies [21, 22, 23, 24, 28, 29, 30, 31, 32, 33] involving a total of 5766 patients who received either remdesivir or molnupiravir were incorporated into the meta‐analysis. All studies included utilized a retrospective design, and each study compared multiple intervention groups. Standardized dosages were used, with molnupiravir administered at 800 mg twice daily and remdesivir delivered through multiple infusions. In all studies, the severity of COVID‐19 was classified as mild to moderate, and all patients were treated as outpatients. Most studies reported a follow‐up duration of 30 days, and the majority were conducted in Italy, reflecting a focused effort to evaluate these treatments within specific healthcare settings. Table 1 provides a comprehensive overview of the key characteristics of the included studies, summarizing critical aspects such as study design, patient demographics, treatment protocols, and outcome measures.

**Table 1:** Main characteristics of retrospective studies included in the systematic review and meta‐analysis.

Study, year	Country	Study design	Severity of COVID‐19	Patient setting	Remdesivir					Molnupiravir					Follow‐up (days)	Key study outcomes
					Mean age	N	Male (%)	SARS‐CoV‐2 vaccination rate b	Comorbidity (%) a	Mean age	N	Male (%)	SARS‐CoV‐2 vaccination rate b	Comorbidity (%) a		
Tibble, 2024 [33]	UK	RS	MM	Outpatients	64	207	57.2	89.3	68.8	78	775	53.9	95.8	38.1	28	Death, hospitalization
Colaneri, 2024 [30]	Italy	RS	MM	Outpatients	66	230	NA	86.1	56.5	78	499	NA	94.8	73.5	NA	Hospitalization, viral clearance
Bai, 2024 [29]	Italy	RS	MM	Outpatients	78	134	58.2	100	100	79	92	64.1	100	100	30	Death, viral clearance, adverse event
Alonso, 2024 [28]	Spain	RS	MM	Outpatients	57.4	34	52.9	NA	NA	62	10	70	NA	NA	NA	Death, hospitalization
Tiseo, 2023 [24]	Italy	RS	MM	Outpatients	NA	20	NA	NA	NA	NA	2910	NA	NA	NA	30	Death, hospitalization, adverse event
Rinaldi, 2023 [23]	Italy	RS	MM	Outpatients	65.7	30	60	23.3	100	68.9	29	62.1	100	100	7	Death, hospitalization, viral clearance, adverse event
Razai, 2023 [32]	Italy	RS	MM	Outpatients	67.4	142	41.6	88	55.6	68.9	205	57.6	88.3	55.1	60	Death, hospitalization
Manciulli, 2023 [22]	Italy	RS	MM	Outpatients	NA	5	20	80	100	NA	7	71.4	71.4	100	28	Death, hospitalization, adverse event
Borgo, 2023 [21]	Italy	RS	MM	Outpatients	78	134	78	91	43	79	92	59	91	51	28	Death, viral clearance, clinical progression, adverse event
Lasagna, 2022 [31]	Italy	RS	MM	Outpatients	65.5	58	55.2	96.6	62.1	79	69	56.5	94.2	81.2	30	Hospitalization, viral clearance

Abbreviations: N, number, NA, not acquired, MM, mild to moderate, RS, retrospective study, a: having at least one comorbidity, b: Receipt of ≥ 1 dose SARS‐CoV‐2 vaccine.

### 3.2 Risk of Bias Assessment and Quality of Evidence

The majority of studies were assessed to have a moderate risk of confounding. Each study exhibited a low risk concerning the classification of interventions and missing data. However, deviations from the intended interventions and the measurement of outcomes were rated as moderate across all studies. The risk associated with other domains varied among the studies. Detailed findings from the ROBINS‐I risk of bias evaluation are presented in Table S1. Additionally, the assessment of evidence certainty for each outcome is provided in Table S2.

### 3.3 Effectiveness Outcomes

#### 3.3.1 Mortality Rate

**Figure 2:**
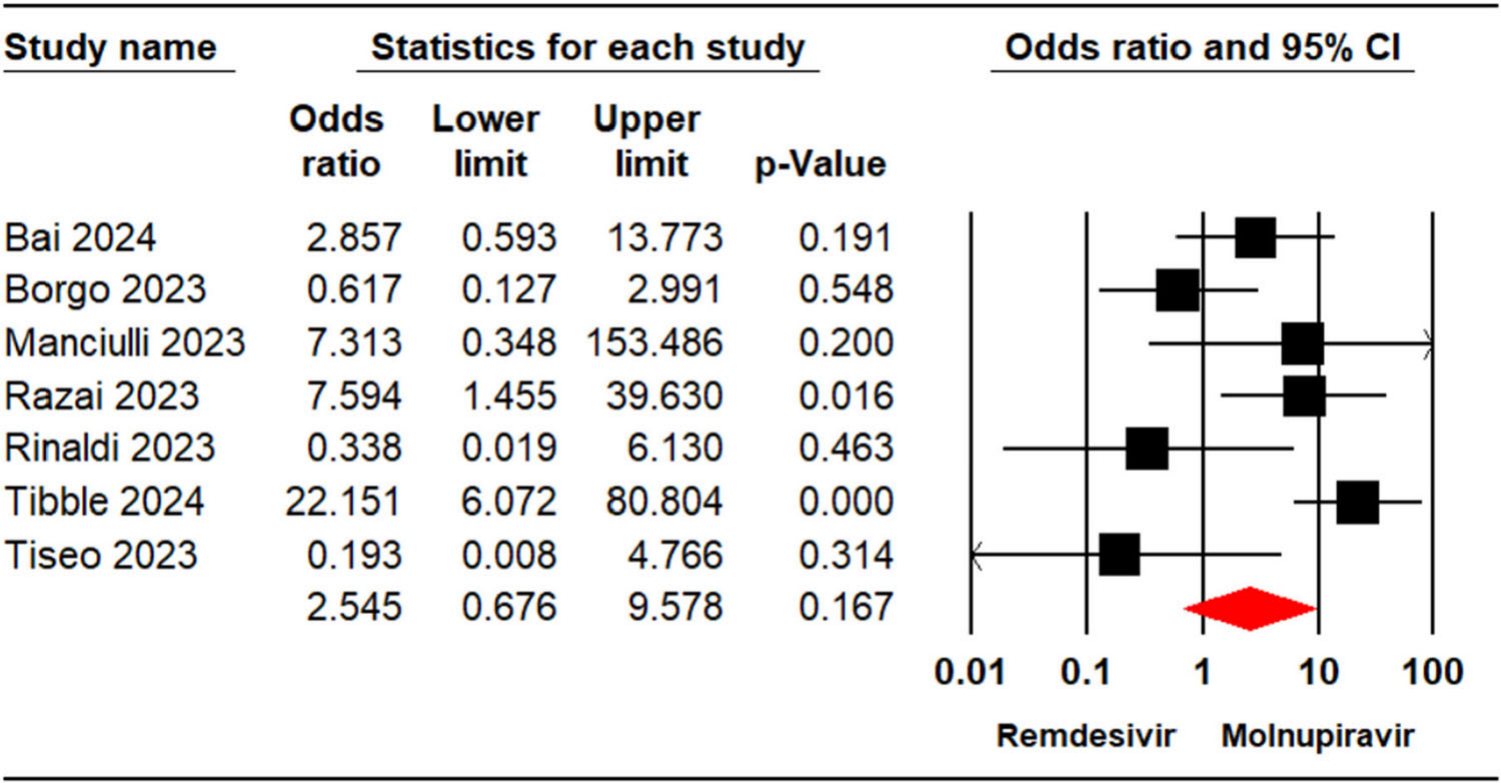
Forest plot illustrating the comparison of mortality rates between remdesivir and molnupiravir.

A total of seven studies [21, 22, 23, 24, 29, 32, 33], involving 5,578 patients, reported mortality outcomes for individuals treated with either remdesivir or molnupiravir. The overall analysis of these studies indicated that there was no significant difference in the mortality rates between the two groups (OR = 2.54, 95% CI: 0.67, 9.57, *p* = 0.16) (Figure 2). Furthermore, subgroup analysis based on follow‐up duration revealed no significant difference in mortality rates between treatments within the < 28 days group (OR = 2.85, 95% CI: 0.59–13.77, *p* = 0.19) and the ≥ 28 days group (OR = 2.36, 95% CI: 0.46–12.11, *p* = 0.30) (Figure S1). The level of certainty regarding the evidence for the mortality outcome was assessed as moderate.

#### 3.3.2 Hospitalization Rate

**Figure 3:**
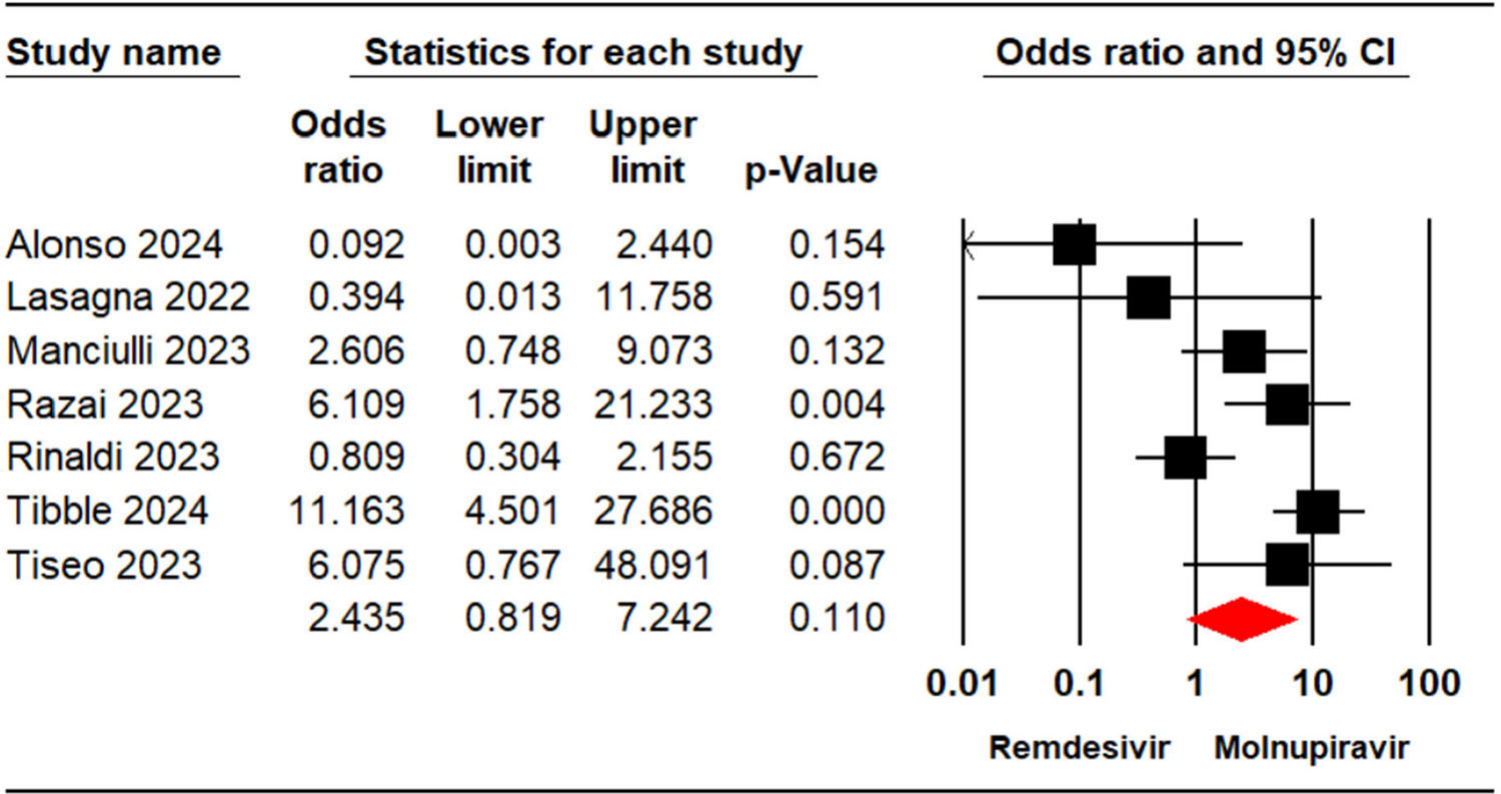
Forest plot illustrating the comparison of hospitalization rates between remdesivir and molnupiravir.

Across seven studies [22, 23, 24, 28, 31, 32, 33] involving 4,679 patients, cases of hospital admission for individuals receiving remdesivir or molnupiravir were documented. The meta‐analysis revealed no significant difference in hospitalization rates between the two treatments (OR = 2.43, 95% CI: 0.81, 7.24, *p* = 0.11) (Figure 3). The level of certainty regarding the evidence for the hospitalization outcome was assessed as moderate.

#### 3.3.3 Viral Clearance Rate

**Figure 4:**
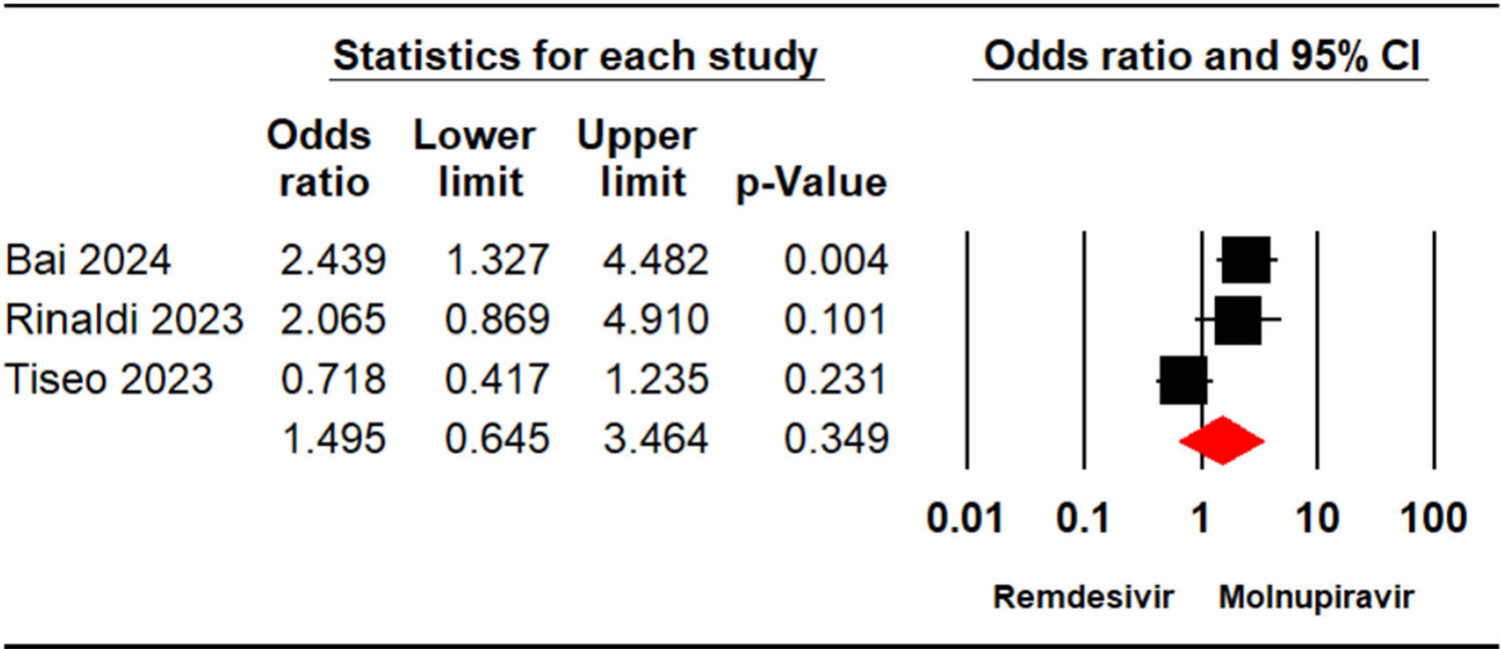
Forest plot illustrating the comparison of viral clearance rates between remdesivir and molnupiravir.

Three studies [23, 24, 29] enrolling a total of 1518 patients reported viral clearance rate in individuals who underwent treatment with remdesivir or molnupiravir. The meta‐analysis demonstrated no significant differences between the two treatments (OR = 1.49, 95% CI: 0.64, 3.46, *p* = 0.34) (Figure 4). The level of certainty regarding the evidence for the viral clearance rate outcome was assessed as low.

#### 3.3.4 Mean Viral Clearance Time

**Figure 5:**
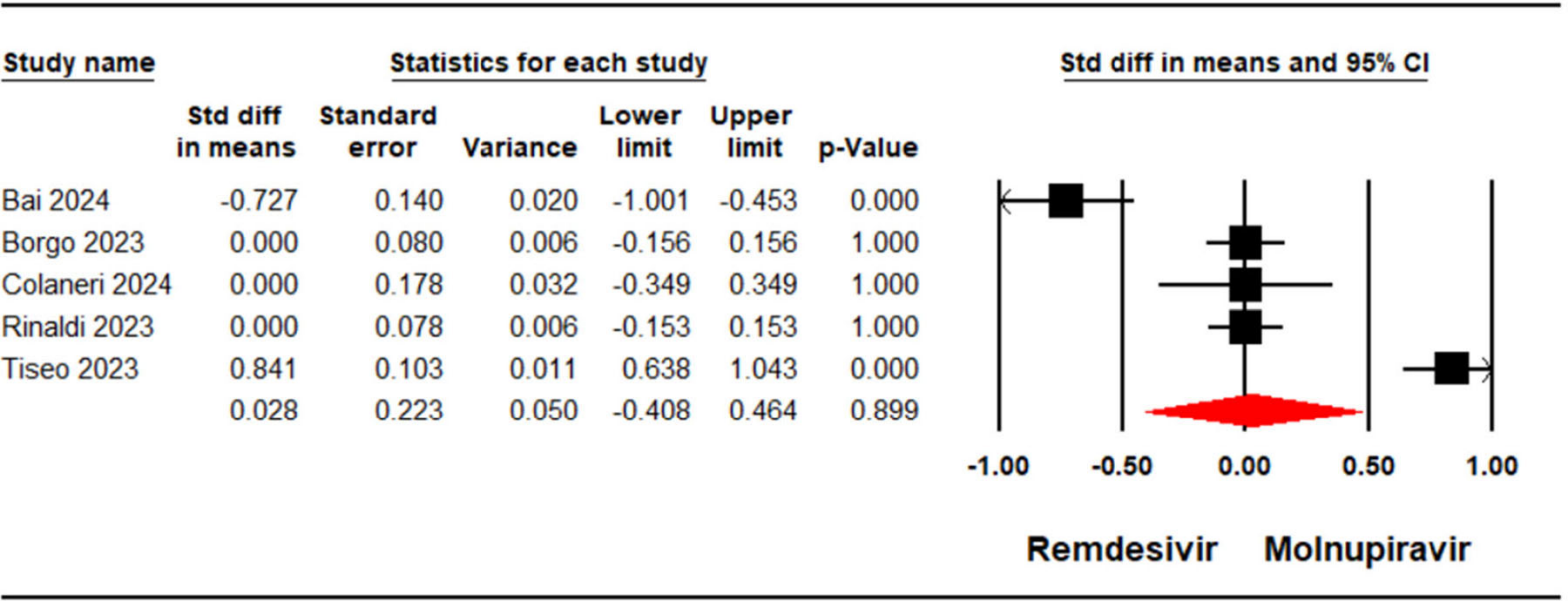
Forest plot illustrating the comparison of mean viral clearance time between remdesivir and molnupiravir.

A meta‐analysis concerning mean viral clearance time, which included five studies [21, 23, 24, 29, 30] encompassing 2474 patients, found no significant difference between individuals receiving remdesivir and those receiving molnupiravir in terms of mean viral clearance (SMD = 0.02, 95% CI: −0.40, 0.46, *p* = 0.89) (Figure 5). The level of certainty regarding the evidence for the mean viral clearance outcome was assessed as low.

### 3.4 Safety Outcomes

#### 3.4.1 Any Adverse Events

**Figure 6:**
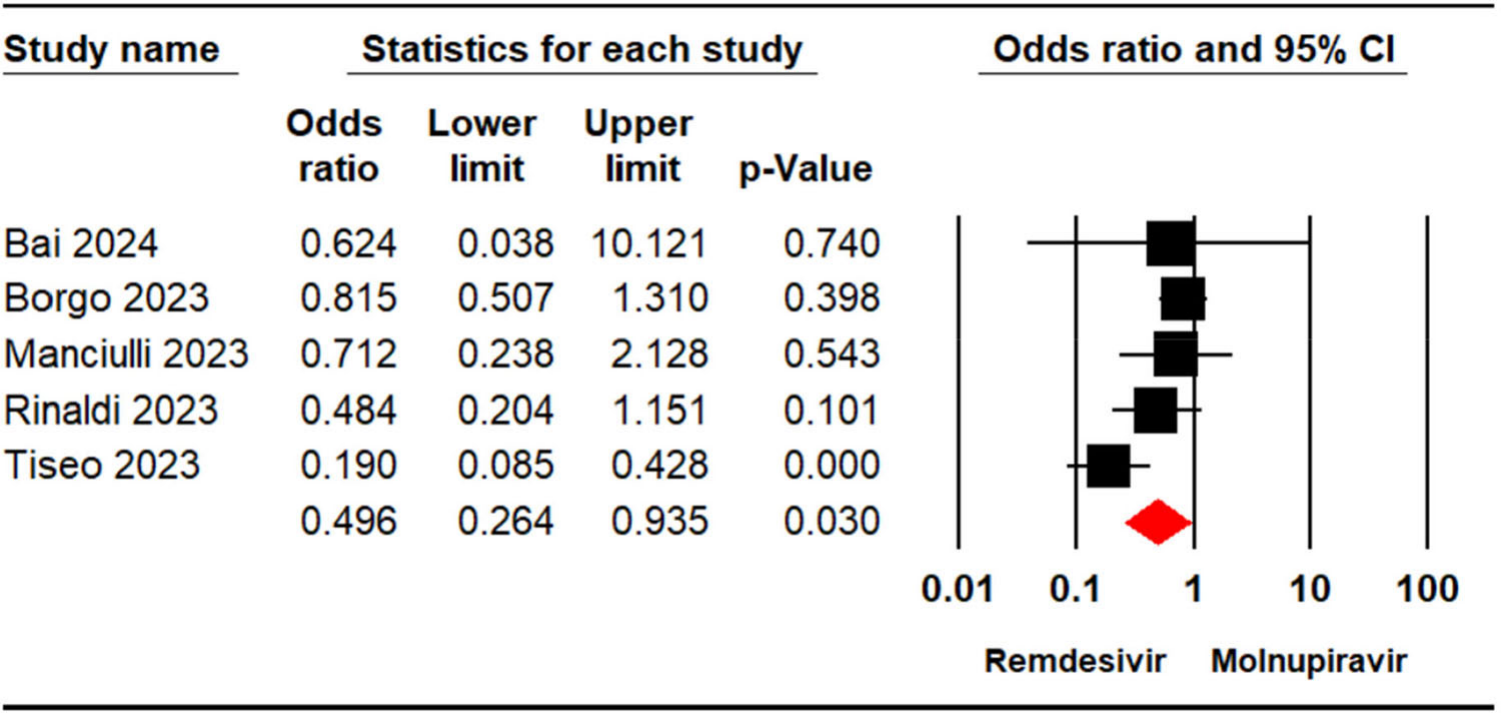
Forest plot illustrating the comparison of adverse events between remdesivir and molnupiravir.

Five studies [21, 22, 23, 24, 29] including 2573 patients detailed the occurrence of any adverse events in the groups receiving remdesivir or molnupiravir. The pooled analysis showed a significant difference in adverse event incidence between the two groups (OR = 0.49, 95% CI: 0.26, 0.93; *p* = 0.03) (Figure 6). The level of certainty regarding the evidence for the adverse events outcome was assessed as moderate.

### 3.5 Sensitivity Analyses

For the outcomes of mortality rate and mean viral clearance time, the leave‐one‐out sensitivity analysis showed no significant changes compared to the primary analysis (Figures S2 and S5). However, the sensitivity analysis revealed significant changes for the outcomes of hospitalization rate, viral clearance rate, and incidence of any adverse events when compared to the primary analysis (Figures S3, S4, and S6). The exclusion of certain studies resulted in notable shifts in the pooled estimates for these outcomes, suggesting that the results may be more sensitive to the inclusion or exclusion of specific studies.

## 4 Discussion

The availability of effective and safe antiviral medications for patients infected with SARS‐CoV‐2, especially those at high risk, is crucial for patient management. These options enable healthcare providers to select the most suitable treatment, potentially improving patient outcomes [34]. This study aimed to evaluate and compare the safety and effectiveness of remdesivir and molnupiravir, two antiviral treatments for COVID‐19, in patients infected with SARS‐CoV‐2. Based on the results of this meta‐analysis, these interventions show no significant difference in improving clinical outcomes in COVID‐19 patients.

The meta‐analysis revealed no statistically significant variation in mortality rates among COVID‐19 patients treated with remdesivir compared to those receiving molnupiravir. This suggests that the two antiviral treatments have similar effects on reducing COVID‐19‐related mortality. Existing evidence on the effectiveness of remdesivir and molnupiravir in lowering mortality has been mixed. While some systematic reviews and meta‐analyses have reported that remdesivir [10, 18, 35] or molnupiravir [19, 36] significantly reduced the risk of death compared to the controls, other analyses found no clinical benefit with remdesivir [37, 38, 39] or molnupiravir [40, 41] in decreasing COVID‐19 mortality. These variations could be due to differences in the quantity of studies included, control group characteristics, and other variables between the meta‐analyses. The comparative effectiveness of molnupiravir versus other antivirals has also varied [24, 42]. According to a meta‐analysis of retrospective studies, the effectiveness of molnupiravir was comparable to that of sotrovimab in improving clinical outcome in COVID‐19 patients [42].

The meta‐analysis revealed no statistically significant difference in hospitalization rates when comparing COVID‐19 patients administered remdesivir to those treated with molnupiravir. However, existing evidence from other studies indicates that both antivirals may effectively reduce the risk of hospitalization when compared to untreated patients. Clinical trial meta‐analyses indicate that molnupiravir treatment is linked to a significant decrease in the likelihood of hospitalization among COVID‐19 patients [19, 20]. These findings indicate that molnupiravir may be effective in preventing severe disease progression requiring hospitalization [19, 20]. Similarly, studies have supported the effectiveness of remdesivir in reducing hospitalization rates among outpatients infected with COVID‐19. For example, Gottlieb et al. [43] reported that only 0.7% of patients treated with remdesivir experienced COVID‐19‐related hospitalization or death, compared to 5.3% in the placebo group. Similarly, a study by Rajme‐López et al. [44] demonstrated that remdesivir treatment significantly reduced the likelihood of hospitalization or mortality. Additionally, Mazzitelli et al. [45] found that patients with COVID‐19 treated with early 3‐day remdesivir had a lower risk of hospitalization compared to controls. Furthermore, remdesivir has been shown to significantly shorten the length of hospital stay for patients hospitalized due to COVID‐19 [46].

The meta‐analysis also found no significant difference in the outcomes of viral clearance rate or mean time to negative PCR between COVID‐19 patients treated with remdesivir and those receiving molnupiravir. However, research indicates that both treatments can significantly reduce the time of SARS‐CoV‐2 positivity in COVID‐19 patients when compared to those who receive no treatment. For example, Mazzitelli et al. [45] found that early administration of remdesivir was significantly associated with a shorter duration of SARS‐CoV‐2 positivity. Similarly, molnupiravir has been shown to effectively shorten the period of SARS‐CoV‐2 positivity when compared to untreated patients [41]. A systematic review and meta‐analysis of randomized controlled demonstrated that the negative PCR rate in COVID‐19 patients treated with Molnupiravir was higher than those who did not receive the drug [40]. Another meta‐analysis of 9 RCTs involving 30,472 adults with mild or moderate COVID‐19 also showed that molnupiravir probably increases the rate of viral clearance compared to those who did not receive the treatment [19]. Retrospective cohort studies comparing remdesivir and molnupiravir found no difference in negative PCR rates between the two treatments [23, 24]. For instance, Rinaldi et al. [23] reported that the negative PCR rate in outpatients with SARS‐CoV‐2 infection was 99.5% in the remdesivir group and 98.1% in the molnupiravir group.

Finally, the meta‐analysis indicated that COVID‐19 patients administered molnupiravir experienced a greater frequency of adverse events than those treated with remdesivir. Systematic reviews and meta‐analyses have shown no significant increase in adverse events for either remdesivir or molnupiravir compared to control groups [37, 41]. For instance, a meta‐analysis involving 31,573 COVID‐19 patients reported no significant difference in adverse events between the molnupiravir and no‐molnupiravir groups [41]. According to the Food and Drug Administration Adverse Event Reporting System (FAERS), the most frequent adverse events and signals for molnupiravir were associated with gastrointestinal and skin or subcutaneous tissue disorders, with patients aged 65 and older showing a heightened risk of cardiac, hepatobiliary, renal, urinary, and vascular disorders [47]. In contrast, FAERS data indicate that remdesivir, as a suspect drug, was linked to disproportionately higher reports of elevated liver enzymes, acute kidney injury, increased blood creatinine levels, bradycardia, cardiac arrest, and death compared to other drugs [48]. Supporting these findings, recent studies reported that remdesivir was associated with hepatotoxicity in 19.5% of patients and acute kidney injury in 17.3%, with older age and SSRI use as risk factors for hepatotoxicity, while male gender was protective; additionally, 37.1% of patients developed bradycardia, with higher risks in those aged ≥ 65 years, with hypertension, or obesity [49, 50]. As for remdesivir, a meta‐analysis found no significant link between its use and a higher risk of adverse effects. Additionally, a study by Santenna et al. [51] demonstrated that remdesivir was a safe antiviral agent for hospitalized COVID‐19 patients compared to controls. When considering severe adverse events, meta‐analyses of clinical trials showed that the incidence of severe adverse events was significantly lower in COVID‐19 patients treated with Remdesivir [10, 18]. Similarly, a meta‐analysis conducted by Tian et al. [41] found comparable results for patients treated with molnupiravir, indicating a favorable safety profile for both antiviral agents. However, close monitoring of safety is essential, particularly for COVID‐19 patients who are elderly or have pre‐existing health conditions, as they may be more susceptible to adverse events. Ongoing surveillance and reporting of adverse events will be crucial to ensure the safe use of these antiviral treatments in clinical practice.

A key consideration in interpreting the outcomes of interest is the difference in disease severity for which molnupiravir and remdesivir are indicated. molnupiravir is specifically authorized for the treatment of mild to moderate COVID‐19 in outpatients at high risk of disease progression [12], whereas remdesivir is primarily used in hospitalized patients with severe COVID‐19, including those requiring supplemental oxygen or mechanical ventilation [13]. However, evidence also supports the efficacy of remdesivir in patients with mild to moderate COVID‐19 [52, 53]. All included studies in the analysis focused on outpatients with mild to moderate COVID‐19, which may introduce heterogeneity due to differences in target populations. The effectiveness and safety profiles of these interventions can vary significantly based on disease severity, underscoring the need for cautious interpretation of the results.

This study has a number of limitations that should be considered. Firstly, the inclusion of retrospective studies in this systematic review and meta‐analysis introduces potential biases that may impact the validity of the findings. Secondly, heterogeneity in COVID‐19 vaccination rates among patients may have influenced the results. Another limitation is the variability in the levels of comorbidities among the study populations, which could affect the comparison of interventions. Thirdly, the predominance of studies conducted in Italy may limit generalizability due to differences in healthcare systems, patient demographics, or SARS‐CoV‐2 variants. Lastly, most studies included in the meta‐analysis had multiple groups, which may have introduced additional complexity in data analysis.

## 5 Conclusion

The meta‐analysis showed no significant difference in outcomes when treating mild to moderate COVID‐19 outpatients with remdesivir compared to molnupiravir. Both interventions showed similar effectiveness in terms of improving outcomes such as mortality rate and hospital admission. The incidence of adverse events was higher in the molnupiravir group. However, it is important to consider two key points when interpreting these findings. Firstly, the evidence supporting these findings was assessed to be of low to moderate certainty. Secondly, these results may not be generalizable to new SARS‐CoV‐2 variants. While these results offer important perspectives on the relative safety and effectiveness of these treatments for COVID‐19, additional high‐quality studies are necessary to validate or challenge these conclusions.

## Author Contributions

**Seyed Hamid Pakzad Moghadam:** conceptualization, formal analysis, project administration, supervision. **Ali Sarkoohi:** investigation, methodology. **Zia Navidi:** investigation, methodology. **Bahman Amani:** investigation, methodology. **Behnam Amani:** investigation, methodology. **Saeed Khorramnia:** conceptualization, formal analysis, project administration, writing – original draft, writing – review and editing.

## Supporting information


**Table S1:** ROBINS‐I tool results for non‐randomized studies. **Table S2:** Assessment of certainty of evidence using the GRADE approach. **Figure S1:** Forest plot showing the results of subgroup analysis comparing mortality rates between remdesivir and molnupiravir. **Figure S2:** Forest plot showing the results of the leave‐one‐out sensitivity analysis for the comparison of mortality rates between remdesivir and molnupiravir. **Figure S3:** Forest plot showing the results of the leave‐one‐out sensitivity analysis for the comparison of hospitalization rates between remdesivir and molnupiravir. **Figure S4:** Forest plot showing the results of the leave‐one‐out sensitivity analysis for the comparison of viral clearance rates between remdesivir and molnupiravir. **Figure S5:** Forest plot showing the results of the leave‐one‐out sensitivity analysis for the comparison of mean viral clearance time between remdesivir and molnupiravir. **Figure S6:** Forest plot showing the results of the leave‐one‐out sensitivity analysis for the comparison of incidence of adverse events between remdesivir and molnupiravir.

PRISMA 2020 checklist.

## Data Availability

Data are available online for the included studies [21–24, 28–33].

## References

[1] S. Calimeri , D. Lo Giudice , A. Buda , et al., “Role of the 1st Booster Dose of COVID‐19 Vaccine in the Protection Against the Infection: A Fundamental Public Health Tool,” Journal of Preventive Medicine and Hygiene 63, no. 4 (2022): 520. 10.15167/2421-4248/jpmh2022.63.4.2742 PMC9986990 36891000

[2] J. A. Clarke , T. L. Wiemken , and K. M. Korenblat , “Excess Mortality Among Solid Organ Transplant Recipients in the United States During the COVID‐19 Pandemic,” Transplantation 106, no. 12 (2022): 2399–2407. 36042551 10.1097/TP.0000000000004341 PMC9696767

[3] L. Matrajt , E. R. Brown , M. S. Cohen , D. Dimitrov , and H. Janes , “Could Widespread Use of Antiviral Treatment Curb the COVID‐19 Pandemic? A Modeling Study,” BMC Infectious Diseases 22, no. 1 (2022): 683. 35945513 10.1186/s12879-022-07639-1 PMC9361252

[4] D. Poddighe and M. Aljofan , “Clinical Evidences on the Antiviral Properties of Macrolide Antibiotics in the COVID‐19 Era and Beyond,” Antiviral Chemistry and Chemotherapy 28 (2020): 2040206620961712. 32972196 10.1177/2040206620961712 PMC7522830

[5] E. Ricciotti , K. Laudanski , and G. A. FitzGerald , “Nonsteroidal Anti‐Inflammatory Drugs and Glucocorticoids in Covid‐19,” Advances in Biological Regulation 81 (2021): 100818. 34303107 10.1016/j.jbior.2021.100818 PMC8280659

[6] D. Poddighe and E. Kovzel , “Impact of Anti‐Type 2 Inflammation Biologic Therapy on COVID‐19 Clinical Course and Outcome,” Journal of Inflammation Research 14 (2021): 6845–6853. 34934335 10.2147/JIR.S345665 PMC8684423

[7] S. Kausar , F. Said Khan , M. Ishaq Mujeeb Ur Rehman , et al., “A Review: Mechanism of Action of Antiviral Drugs,” International Journal of Immunopathology and Pharmacology 35 (2021): 20587384211002621. 33726557 10.1177/20587384211002621 PMC7975490

[8] B. Amani and B. Amani , “Comparison of Effectiveness and Safety of Nirmatrelvir/Ritonavir Versus Sotrovimab for COVID‐19: A Systematic Review and Meta‐Analysis,” Expert Review of Anti‐infective Therapy 22, no. 7 (2024): 547–555. 38457124 10.1080/14787210.2024.2326561

[9] B. Amani and B. Amani , “Azvudine Versus Paxlovid in COVID‐19: A Systematic Review and Meta‐Analysis,” Reviews in Medical Virology 34, no. 4 (2024): e2551. 38849982 10.1002/rmv.2551

[10] M. T. Angamo , M. A. Mohammed , and G. M. Peterson , “Efficacy and Safety of Remdesivir in Hospitalised COVID‐19 Patients: A Systematic Review and Meta‐Analysis,” Infection 50, no. 1 (2022): 27–41. 34331674 10.1007/s15010-021-01671-0 PMC8325414

[11] K. M. Souza , G. Carrasco , R. Rojas‐Cortés , et al., “Effectiveness of Nirmatrelvir‐Ritonavir for the Treatment of Patients With Mild to Moderate COVID‐19 and at High Risk of Hospitalization: Systematic Review and Meta‐Analyses of Observational Studies,” PLoS One 18, no. 10 (2023): e0284006. 37824507 10.1371/journal.pone.0284006 PMC10569636

[12] https://www.fda.gov/news-events/press-announcements/coronavirus-covid-19-update-fda-authorizes-additional-oral-antiviral-treatment-covid-19-certain.

[13] https://www.fda.gov/news-events/press-announcements/fda-approves-first-treatment-covid-19.

[14] World Health Organization . Molnupiravir for Non‐Severe COVID‐19. Geneva: World Health, https://iris.who.int/server/api/core/bitstreams/94c83217-2d97-42fe-aa34-52b76dfcfaab/content.

[15] COVID‐19 Treatment Guidelines Panel . COVID‐19 treatment guidelines [Internet]. Bethesda (MD): National Institutes of Health; 2023. https://www.ncbi.nlm.nih.gov/books/NBK570371/pdf/Bookshelf_NBK570371.pdf. 34003615

[16] Infectious Diseases Society of America . IDSA Guidelines on the Treatment and Management of Patients With COVID‐19 [Internet]. Arlington (VA): Infectious Diseases Society of America; 2023, https://www.idsociety.org/practice-guideline/covid-19-guideline-treatment-and-management/.

[17] A. Al‐Abdouh , A. Bizanti , M. Barbarawi , et al., “Remdesivir for the Treatment of COVID‐19: A Systematic Review and Meta‐Analysis of Randomized Controlled Trials,” Contemporary Clinical Trials 101 (2021): 106272. 33422642 10.1016/j.cct.2021.106272 PMC7789823

[18] H. K. Elsawah , M. A. Elsokary , M. S. Abdallah , and A. H. ElShafie , “Efficacy and Safety of Remdesivir in Hospitalized Covid‐19 Patients: Systematic Review and Meta‐Analysis Including Network Meta‐Analysis,” Reviews in Medical Virology 31, no. 4 (2021): e2187. 33128490 10.1002/rmv.2187

[19] Y. Gao , M. Liu , Z. Li , J. Xu , J. Zhang , and J. Tian , “Molnupiravir for Treatment of Adults With Mild or Moderate COVID‐19: A Systematic Review and Meta‐Analysis of Randomised Controlled Trials,” Clinical Microbiology and Infection 29, no. 8 (2023): 979–999. 37084941 10.1016/j.cmi.2023.04.014 PMC10116122

[20] M. Sun , H. Lai , J. Huang , et al., “Molnupiravir for the Treatment of Non‐Severe COVID‐19: A Systematic Review and Meta‐Analysis of 14 Randomized Trials With 34 570 Patients,” Journal of Antimicrobial Chemotherapy 78, no. 9 (2023): 2131–2139. 37437106 10.1093/jac/dkad216

[21] C. Del Borgo , S. Garattini , C. Bortignon , et al., “Effectiveness, Tolerability and Prescribing Choice of Antiviral Molecules Molnupiravir, Remdesivir and Nirmatrelvir/R: A Real‐World Comparison in the First Ten Months of Use,” Viruses 15, no. 4 (2023): 1025. 37113006 10.3390/v15041025 PMC10145588

[22] T. Manciulli , M. Spinicci , B. Rossetti , et al., “Safety and Efficacy of Outpatient Treatments for COVID‐19: Real‐Life Data From a Regionwide Cohort of High‐Risk Patients in Tuscany, Italy (The Federate Cohort),” Viruses 15, no. 2 (2023): 438. 36851654 10.3390/v15020438 PMC9967010

[23] M. Rinaldi , C. Campoli , M. Gallo , et al., “Comparison Between Available Early Antiviral Treatments in Outpatients With SARS‐CoV‐2 Infection: A Real‐Life Study,” BMC Infectious Diseases 23, no. 1 (2023): 646. 37784051 10.1186/s12879-023-08538-9 PMC10546723

[24] G. Tiseo , C. Barbieri , V. Galfo , et al., “Efficacy and Safety of Nirmatrelvir/Ritonavir, Molnupiravir, and Remdesivir in a Real‐World Cohort of Outpatients With COVID‐19 at High Risk of Progression: The PISA Outpatient Clinic Experience,” Infectious Diseases and Therapy 12, no. 1 (2023): 257–271. 36441485 10.1007/s40121-022-00729-2 PMC9707131

[25] M. J. Page , J. E. McKenzie , P. M. Bossuyt , et al., “The Prisma 2020 Statement: An Updated Guideline for Reporting Systematic Reviews,” BMJ 372 (2021): n71. 10.1136/bmj.n71. 33782057 PMC8005924

[26] J. A. Sterne , M. A. Hernán , B. C. Reeves , et al, “Robins‐I: A Tool for Assessing Risk of Bias in Non‐Randomised Studies of Interventions,” BMJ (2016): 355. 10.1136/bmj.i4919 PMC5062054 27733354

[27] A. Granholm , W. Alhazzani , and M. H. Møller , “Use of the Grade Approach in Systematic Reviews and Guidelines,” British Journal of Anaesthesia 123, no. 5 (2019): 554–559. 31558313 10.1016/j.bja.2019.08.015

[28] M. Alonso , F. Villanego , L. A. Vigara , et al., “Real‐World Experience With Mild‐Moderate COVID‐19 Therapies in Kidney Transplant Patients: How to Treat Patients With Chronic Kidney Disease From Now On?,” Nefrología (English Edition) 44 (2024): 433–435. 10.1016/j.nefroe.2024.06.003 38918164

[29] F. Bai , T. Beringheli , V. Vitaletti , et al., “Clinical Outcome and 7‐Day Virological Clearance in High‐Risk Patients With Mild–Moderate COVID‐19 Treated With Molnupiravir, Nirmatrelvir/Ritonavir, or Remdesivir,” Infectious Diseases and Therapy 13 (2024): 1589–1605. 38829439 10.1007/s40121-024-00994-3 PMC11219607

[30] M. Colaneri , G. Scaglione , F. Fassio , et al., “Early Administration of Nirmatrelvir/Ritonavir Leads to Faster Negative SARS‐CoV‐2 Nasal Swabs Than Monoclonal Antibodies in Covid 19 Patients at High‐Risk for Severe Disease,” Virology Journal 21, no. 1 (2024): 68. 38509536 10.1186/s12985-024-02333-x PMC10953281

[31] A. Lasagna , I. Cassaniti , D. Lilleri , et al., “Effectiveness of the Available Early Therapies in Reducing Severe COVID‐19 in Non‐Hospitalized Patients With Solid Tumors on Active Treatment,” Frontiers in Medicine 9 (2022): 1036473. 36388947 10.3389/fmed.2022.1036473 PMC9643502

[32] D. Razia , D. Sindu , K. Grief , et al., “349) Molnupiravir vs Remdesivir for Treatment of Covid‐19 in Lung Transplant Recipients,” Journal of Heart and Lung Transplantation 42, no. 4 (2023): S165–S166.

[33] H. Tibble , T. Mueller , E. Proud , et al., “Real‐World Severe COVID‐19 Outcomes Associated With Use of Antivirals and Neutralising Monoclonal Antibodies in Scotland,” NPJ Primary Care Respiratory Medicine 34, no. 1 (2024): 17. 10.1038/s41533-024-00374-x PMC11213868 38942748

[34] B. Amani and B. Amani , “Effectiveness and Safety of Azvudine in COVID‐19: A Systematic Review and Meta‐Analysis,” PLoS One 19, no. 6 (2024): e0298772. 38870134 10.1371/journal.pone.0298772 PMC11175417

[35] V. Bansal , K. S. Mahapure , A. Bhurwal , et al., “Mortality Benefit of Remdesivir in COVID‐19: A Systematic Review and Meta‐Analysis,” Frontiers in Medicine 7 (2021): 606429. 33585508 10.3389/fmed.2020.606429 PMC7873594

[36] H. A. Cheema , S. Abdul Rab , M. Butt , et al., “Molnupiravir for the Treatment of COVID‐19 Outpatients: An Updated Meta‐Analysis,” Journal of Microbiology, Immunology and Infection 57 (2024): 396–402. 10.1016/j.jmii.2024.03.002 38555274

[37] C.‐C. Lai , C.‐H. Chen , C.‐Y. Wang , K.‐H. Chen , Y.‐H. Wang , and P.‐R. Hsueh , “Clinical Efficacy and Safety of Remdesivir in Patients With COVID‐19: a Systematic Review and Network Meta‐Analysis of Randomized Controlled Trials,” Journal of Antimicrobial Chemotherapy 76, no. 8 (2021): 1962–1968. 33758946 10.1093/jac/dkab093 PMC8083728

[38] G. N. Okoli , R. Rabbani , L. Copstein , A. Al‐Juboori , N. Askin , and A. M. Abou‐Setta , “Remdesivir for Coronavirus Disease 2019 (COVID‐19): A Systematic Review With Meta‐Analysis and Trial Sequential Analysis of Randomized Controlled Trials,” Infectious Diseases 53, no. 9 (2021): 691–699. 33974479 10.1080/23744235.2021.1923799 PMC8127173

[39] A. Piscoya , L. F. Ng‐Sueng , A. Parra del Riego , et al., “Efficacy and Harms of Remdesivir for the Treatment of COVID‐19: A Systematic Review and Meta‐Analysis,” PLoS One 15, no. 12 (2020): e0243705. 33301514 10.1371/journal.pone.0243705 PMC7728272

[40] P. Y. Huang , T. H. Liu , J. Y. Wu , Y. W. Tsai , and C. C. Lai , “Clinical Efficacy and Safety of Molnupiravir for Nonhospitalized and Hospitalized Patients With COVID‐19: A Systematic Review and Meta‐Analysis of Randomized Control Trials,” Journal of Medical Virology 95, no. 3 (2023): e28621. 36846901 10.1002/jmv.28621

[41] F. Tian , Q. Feng , and Z. Chen , “Efficacy and Safety of Molnupiravir Treatment for COVID‐19: a Systematic Review and Meta‐Analysis of Randomized Controlled Trials,” International Journal of Antimicrobial Agents 62 (2023): 106870. 37245600 10.1016/j.ijantimicag.2023.106870 PMC10214763

[42] B. Amani and B. Amani , “Comparison of Effectiveness and Safety of Molnupiravir Versus Sotrovimab for COVID‐19: A Systematic Review and Meta‐Analysis,” Immunity, Inflammation and Disease 12, no. 4 (2024): e1262. 38652021 10.1002/iid3.1262 PMC11037253

[43] R. L. Gottlieb , C. E. Vaca , R. Paredes , et al., “Early Remdesivir to Prevent Progression to Severe Covid‐19 in Outpatients,” New England Journal of Medicine 386, no. 4 (2022): 305–315. 34937145 10.1056/NEJMoa2116846 PMC8757570

[44] S. Rajme‐López , B. A. Martinez‐Guerra , J. Zalapa‐Soto , et al., Early Outpatient Treatment With Remdesivir in Patients at High Risk for Severe COVID‐19: A Prospective Cohort Study, Vol. 9 (Oxford University Press US, 2022), ofac502. 10.1093/ofid/ofac502 PMC9585545 36285176

[45] M. Mazzitelli , M. Trunfio , L. Sasset , et al., “Risk of Hospitalization and Sequelae in Patients With COVID‐19 Treated With 3‐Day Early Remdesivir vs. Controls in the Vaccine and Omicron Era: A Real‐Life Cohort Study,” Journal of Medical Virology 95, no. 3 (2023): e28660. 36905216 10.1002/jmv.28660

[46] A. Libra , N. Ciancio , G. Sambataro , et al., “Use of Remdesivir in Patients Hospitalized for COVID‐19 Pneumonia: Effect on the Hypoxic and Inflammatory State,” Viruses 15, no. 10 (2023): 2101. 37896877 10.3390/v15102101 PMC10612076

[47] Y. Liang , L. Ma , Y. Wang , J. Zheng , L. Su , and J. Lyu , “Adverse Events Associated With Molnupiravir: A Real‐World Disproportionality Analysis in Food and Drug Administration Adverse Event Reporting System,” Frontiers in Pharmacology 14 (2023): 1253799. 38026949 10.3389/fphar.2023.1253799 PMC10644225

[48] A. Singh and A. Kamath , “Assessment of Adverse Events Associated With Remdesivir Use for Coronavirus Disease 2019 Using Real‐World Data,” Expert Opinion on Drug Safety 20, no. 12 (2021): 1559–1564. 34328807 10.1080/14740338.2021.1962846

[49] Y. S. Alsowaida , S. A. Alowais , D. Alsowaida , et al., “Frequency, Grades of Toxicity, and Predictors of Hepatotoxicity and Acute Kidney Injury With Remdesivir in COVID‐19 Patients: A Multicenter Retrospective Cohort Study,” Healthcare 13 (2025): 2143. 40941495 10.3390/healthcare13172143 PMC12428545

[50] Y. S. Alsowaida , F. Shehadeh , M. Kalligeros , and E. Mylonakis , “Incidence and Potential Risk Factors for Remdesivir‐Associated Bradycardia in Hospitalized Patients With COVID‐19: A Retrospective Cohort Study,” Frontiers in Pharmacology 14 (2023): 1106044. 36817161 10.3389/fphar.2023.1106044 PMC9930471

[51] C. Santenna , K. Vidyasagar , K. C. Amarneni , et al., “The Safety, Tolerability and Mortality Reduction Efficacy of Remdesivir; Based on Randomized Clinical Trials, Observational and Case Studies Reported Safety Outcomes: An Updated Systematic Review and Meta‐Analysis,” Therapeutic Advances in Drug Safety 12 (2021): 20420986211042517. 34594487 10.1177/20420986211042517 PMC8477695

[52] M. Falcone , L. R. Suardi , G. Tiseo , et al., “Early Use of Remdesivir and Risk of Disease Progression in Hospitalized Patients With Mild to Moderate Covid‐19,” Clinical Therapeutics 44, no. 3 (2022): 364–373. 35120742 10.1016/j.clinthera.2022.01.007 PMC8761549

[53] B.‐H. Ryu , J. Y. Lee , and S. H. Lee , “The Effect of Early Versus Late Remdesivir Treatment in Hospitalized Mild to Moderate COVID‐19 Patients in the Omicron Era: A Retrospective Study,” Medicine 103, no. 29 (2024): e39035. 39029053 10.1097/MD.0000000000039035 PMC11398828

