# Supplementary material for: Comparing the Effectiveness and Safety of Remdesivir and Molnupiravir in COVID‐19: A Systematic Review and Meta‐Analysis
Source: Immun Inflamm Dis. 2025 Oct 16;13(10):e70289. doi: 10.1002/iid3.70289 (PMC12531351; doi:10.1002/iid3.70289)
Supplement: Supplementary file 1 — Table S1: ROBINS‐I tool results for non‐randomized studies. Table S2: Assessment of certainty of evidence using the GRADE approach. Figure S1: Forest plot showing the results of subgroup analysis comparing mortality rates between remdesivir and molnupiravir. Figure S2: Forest plot showing the results of the leave‐one‐out sensitivity analysis for the comparison of mortality rates between remdesivir and molnupiravir. Figure S3: Forest plot showing the results of the leave‐one‐out sensitivity analysis for the comparison of hospitalization rates between remdesivir and molnupiravir. Figure S4: Forest plot showing the results of the leave‐one‐out sensitivity analysis for the comparison of viral clearance rates between remdesivir and molnupiravir. Figure S5: Forest plot showing the results of the leave‐one‐out sensitivity analysis for the comparison of mean viral clearance time between remdesivir and molnupiravir. Figure S6: Forest plot showing the results of the leave‐one‐out sensitivity analysis for the comparison of incidence of adverse events between remdesivir and molnupiravir. [file IID3-13-e70289-s002.docx]

Supplementary

Table S1 - ROBINS-I tool results for non-randomized studies

| Study | Confounding | Selection | Classification of interventions | deviations from intended interventions | Missing Data | Measurement  of outcomes | Reported  Result | Overall |
| --- | --- | --- | --- | --- | --- | --- | --- | --- |
| Alonso, 2024 | Serious | Serious | Moderate | Moderate | Low | Moderate | Moderate | Moderate |
| Bai, 2024 | Low | Low | Moderate | Moderate | Low | Moderate | Low | Moderate |
| Borgo, 2023 | Moderate | Moderate | Moderate | Moderate | Low | Moderate | Moderate | Moderate |
| Colaneri, 2024 | Moderate | Moderate | Moderate | Moderate | Low | Moderate | Moderate | Moderate |
| Lasagna, 2022 | Moderate | Moderate | Moderate | Moderate | Low | Moderate | Moderate | Moderate |
| Manciulli, 2023 | Moderate | Moderate | Moderate | Moderate | Low | Moderate | Moderate | Moderate |
| Razai, 2023 | Moderate | Moderate | Moderate | Moderate | Low | Moderate | Moderate | Moderate |
| Rinaldi, 2023 | Low | Low | Moderate | Moderate | Low | Moderate | Low | Moderate |
| Tibble, 2024 | Low | Low | Moderate | Moderate | Low | Moderate | Low | Moderate |
| Tiseo, 2023 | Moderate | Moderate | Moderate | Moderate | Low | Moderate | Low | Moderate |

Note: Moderate= the study is sound for a non-randomized study with regard to this domain but cannot be considered comparable to a well-performed randomized trial; Low=the study is comparable to a well-performed randomized trial with regard to this domain; Serious= risk of bias =the study has some important problems

Table S2 Assessment of certainty of evidence using the GRADE approach

| Certainty assessment | | | | | | | Effect | Certainty |
| --- | --- | --- | --- | --- | --- | --- | --- | --- |
| No. of studies | Study design | Risk of bias | Inconsistency | Indirectness | Imprecision | Other | Point estimate  (95% CI) |  |
| Mortality rate | | | | | | | | |
| 7 | RS | Serious | Serious | Not serious | Serious | None | 2.54 (0.67, 9.57) | Moderate |
| Hospitalization rate | | | | | | | | |
| 7 | RS | Serious | Serious | Not serious | Serious | None | 2.43 (0.89, 7.24) | Moderate |
| Viral clearance rate | | | | | | | | |
| 3 | RS | Serious | Very Serious | Not serious | Serious | None | 1.15 (0.41, 3.22) | Low |
| Mean viral clearance time | | | | | | | | |
| 5 | RS | Serious | Very Serious | Not serious | Serious | None | 0.28 (-0.40, 0.46) | Low |
| Adverse events | | | | | | | | |
| 5 | RS | Serious | Serious | Not serious | Serious | None | 0.49 (0.26, 0.93) | Moderate |

Low: Our confidence in the effect estimate is limited: The true effect may be substantially different from the estimate of the effect.

Moderate: We are moderately confident in the effect estimate: The true effect is likely to be close to the estimate of the effect, but there is a possibility that it is substantially different

**Figures**


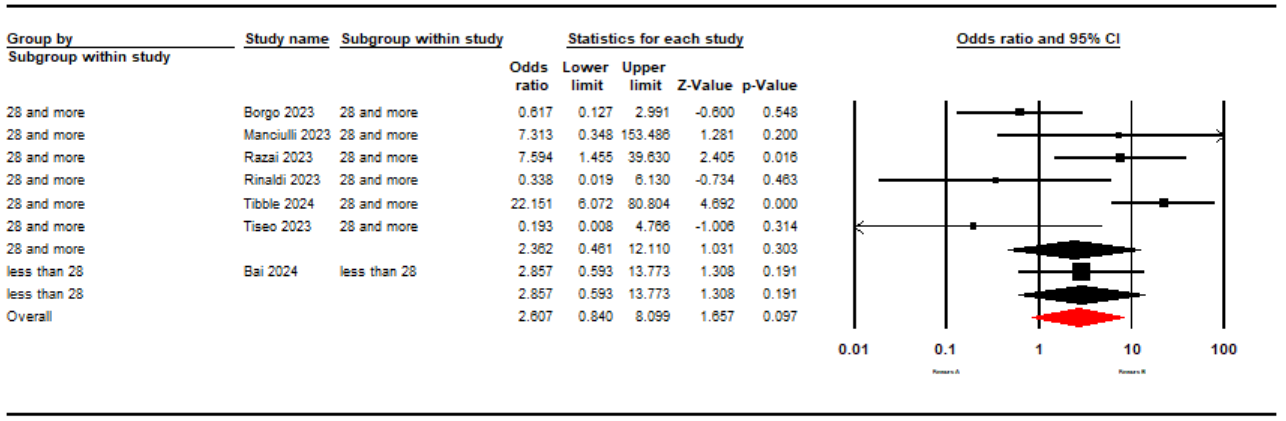


Figure S1 Forest plot showing the results of subgroup analysis comparing mortality rates between remdesivir and molnupiravir


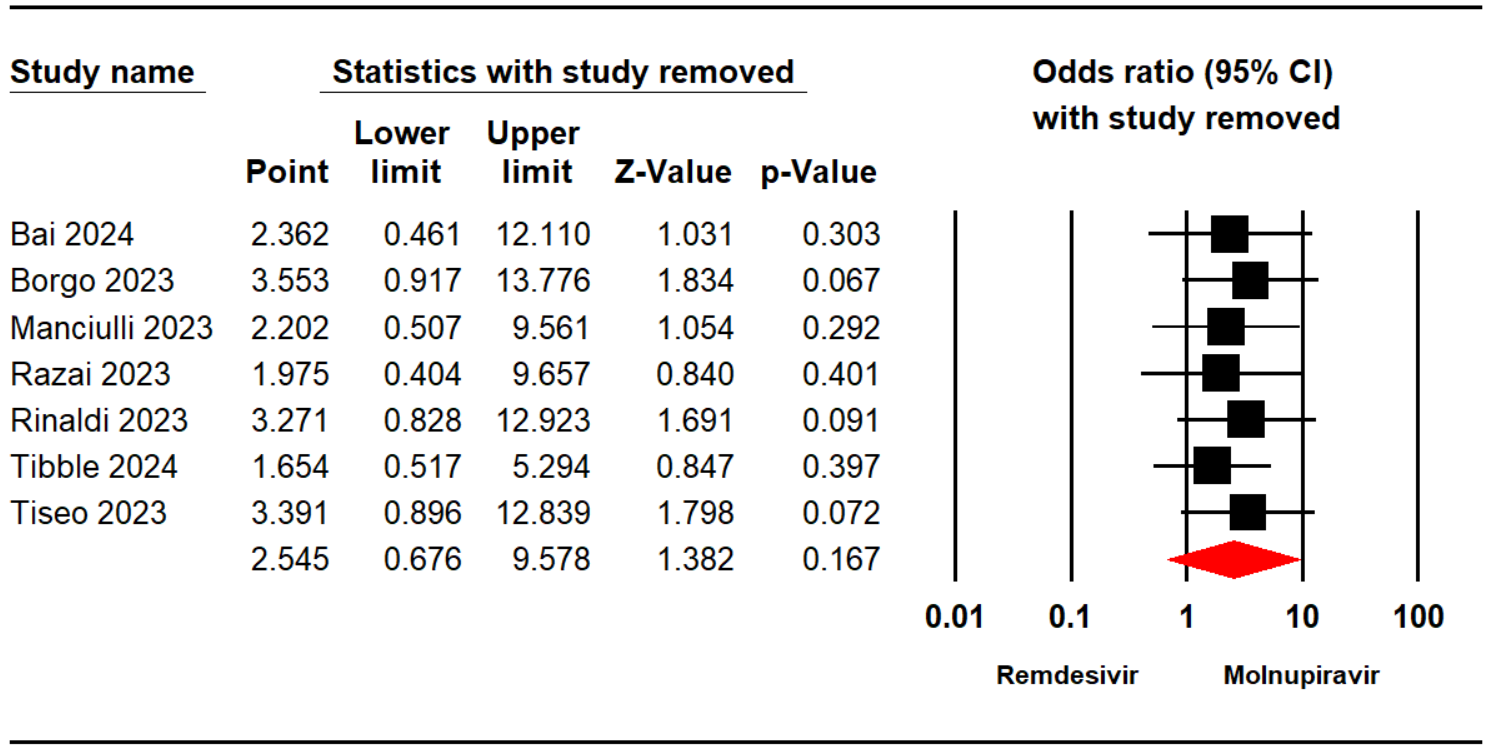


Figure S2 Forest plot showing the results of the leave-one-out sensitivity analysis for the comparison of mortality rates between remdesivir and molnupiravir


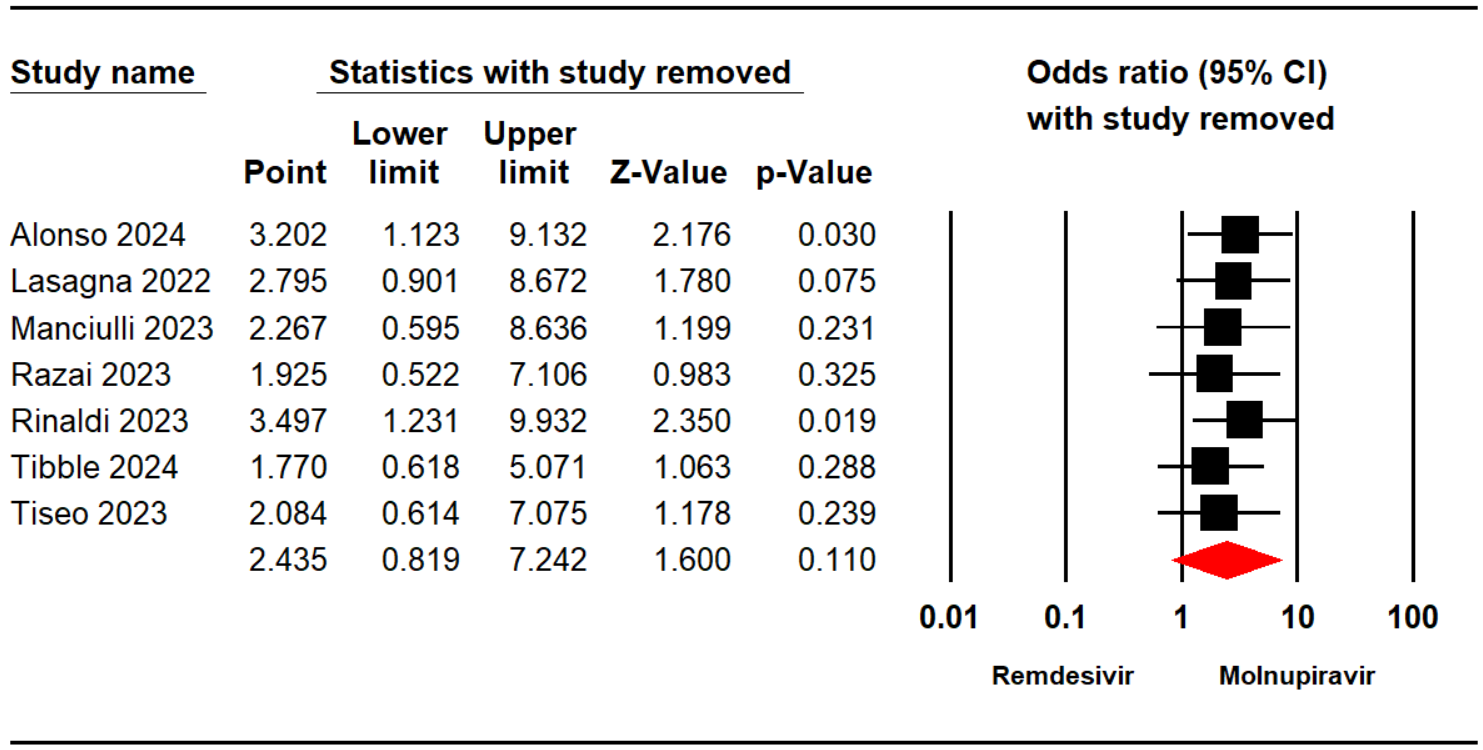


Figure S3 Forest plot showing the results of the leave-one-out sensitivity analysis for the comparison of hospitalization rates between remdesivir and molnupiravir


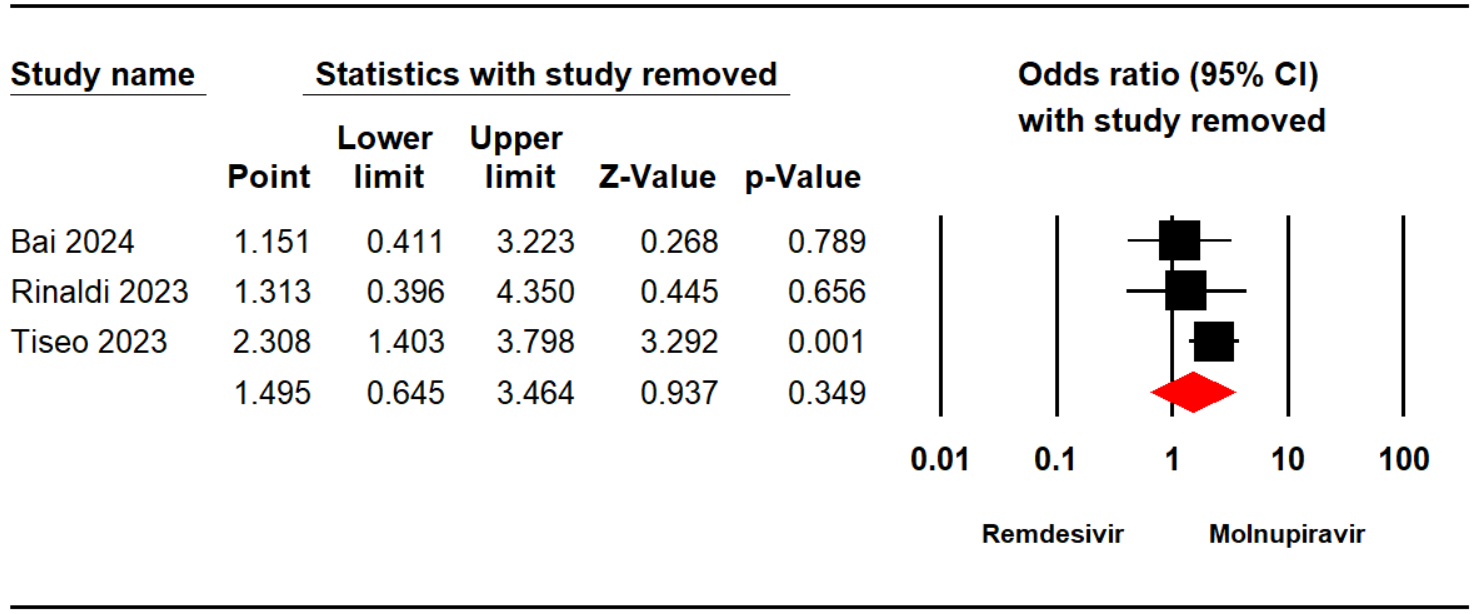


Figure S4 Forest plot showing the results of the leave-one-out sensitivity analysis for the comparison of viral clearance rates between remdesivir and molnupiravir


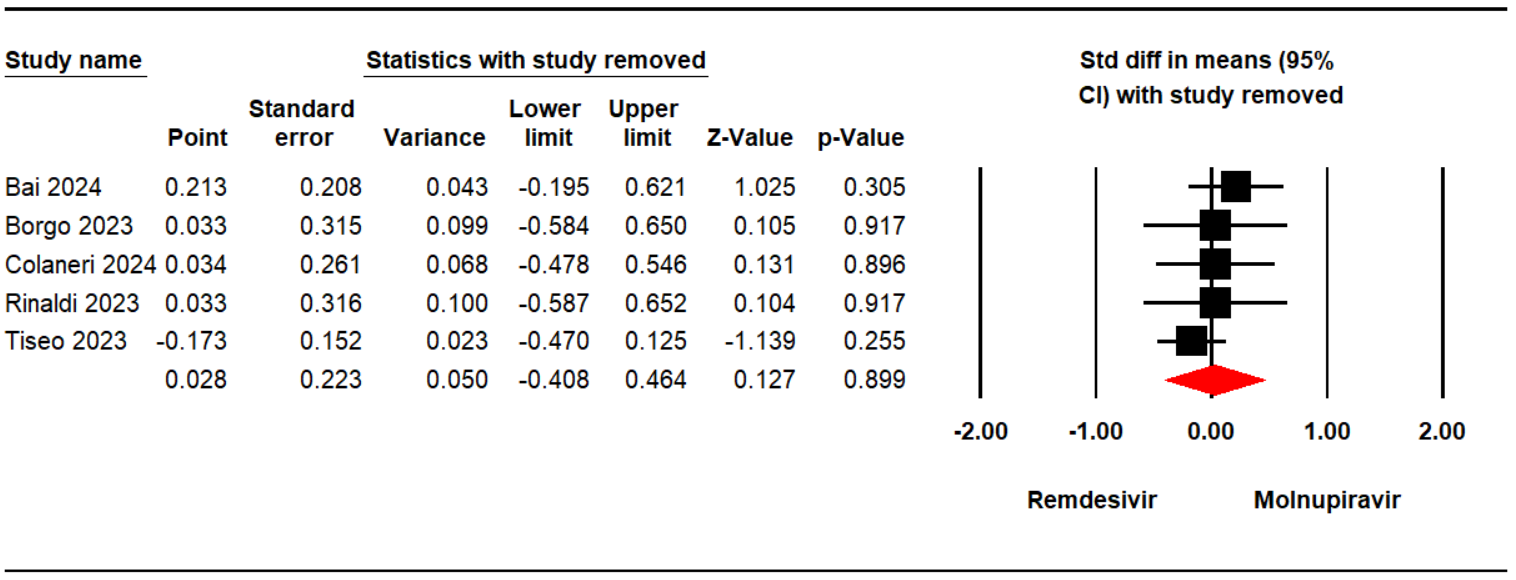


Figure S5 Forest plot showing the results of the leave-one-out sensitivity analysis for the comparison of mean viral clearance time between remdesivir and molnupiravir


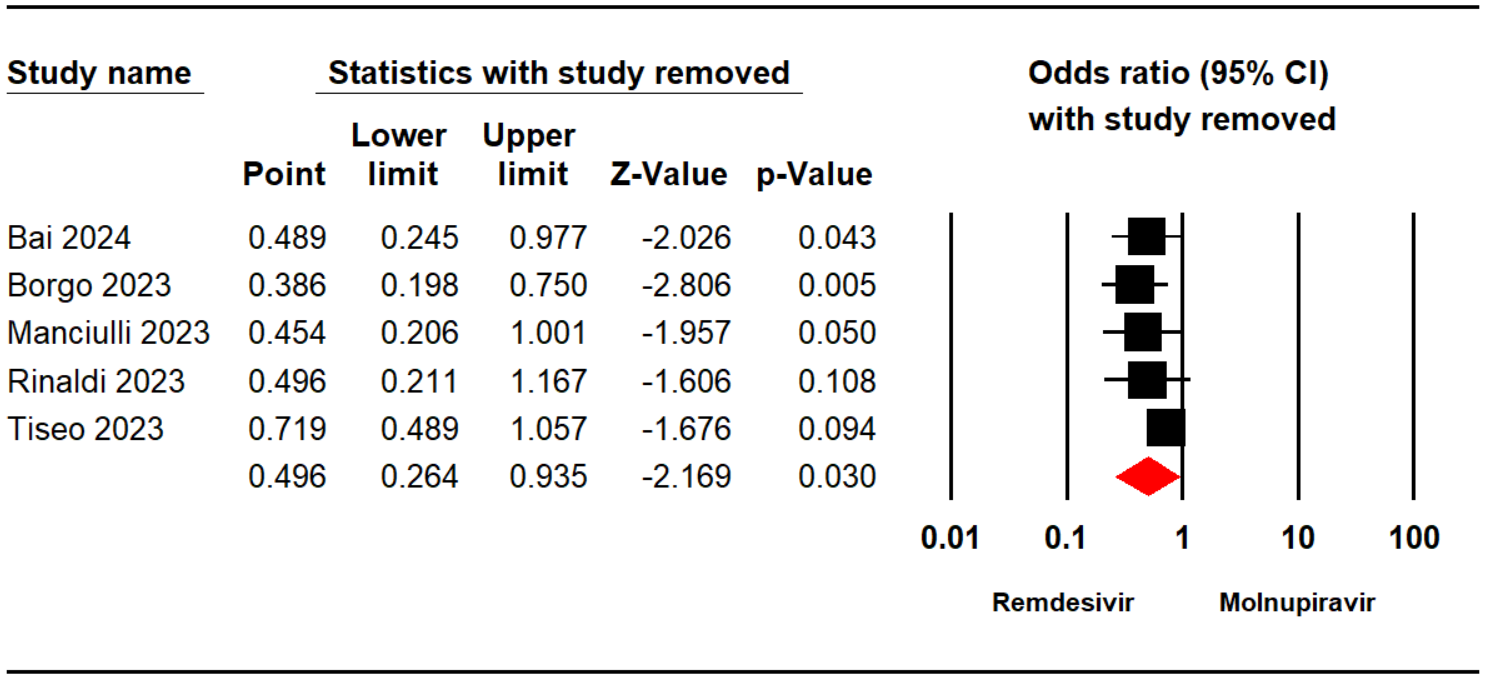


Figure S6 Forest plot showing the results of the leave-one-out sensitivity analysis for the comparison of incidence of adverse events between remdesivir and molnupiravir

**Search strategy**

| **S. No.** | **Database** | **Search String** | **Accessed Date** |
| --- | --- | --- | --- |
| 1 | Cochrane Library | MeSH descriptor: [Coronavirus] explode all trees OR MeSH descriptor: [SARS-CoV-2] explode all trees OR ("coronavirus"):ti,ab,kw OR (COVID-19):ti,ab,kw OR ("coronavirus infection"):ti,ab,kw OR (2019 nCoV):ti,ab,kw OR (2019nCoV):ti,ab,kw OR (nCov 2019):ti,ab,kw OR (SARS CoV2):ti,ab,kw OR (SARS CoV 2):ti,ab,kw OR (SARSCoV2):ti,ab,kw OR (SARSCoV 2):ti,ab,kw OR (severe acute respiratory syndrome coronavirus 2):ti,ab,kw AND " Remdesivir" OR " Veklury" OR " GS-5734" OR " GS5734" OR " RDV" AND "molnupiravir" OR "lagevrio" OR "MK-4482" OR "MK4482" OR " EIDD-2801" | 1 July 2024 |
| 2 | PubMed | #1 (COVID‐19[MeSH Terms]) OR (Coronavirus[MeSH Terms])) OR (SARS‐CoV‐2[MeSH Terms])) OR (coronavirus[Title/Abstract])) OR (COVID-19[Title/Abstract])) OR (coronavirus infection[Title/Abstract])) OR (2019 nCoV[Title/Abstract])) OR (2019nCoV[Title/Abstract])) OR (nCov 2019[Title/Abstract])) OR (SARS CoV2[Title/Abstract])) OR (SARS CoV 2[Title/Abstract])) OR (SARSCoV2[Title/Abstract])) OR (SARSCoV 2[Title/Abstract])) OR (severe acute respiratory syndrome coronavirus 2[Title/Abstract])) OR (novel corona virus disease[Title/Abstract])) OR (corona virus disease 2019[Title/Abstract])) OR (coronavirus disease 2019[Title/Abstract])) OR (novel coronavirus pneumonia[Title/Abstract])) OR (novel corona virus pneumonia[Title/Abstract])  #2 (Remdesivir OR Veklury OR GS-5734 OR GS5734 OR RDV AND molnupiravir OR lagevrio OR MK-4482 OR MK4482 OR EIDD-2801)  #3 #1 AND #2 | 1 July 2024 |
| 3 | Web of Science | TS= (Coronavirus OR "COVID-19" OR "COVID OR COVID19" OR "SARS‐CoV2" OR "SARS-CoV-2" OR SARSCoV2 OR "SARSCoV‐2" OR "SARS coronavirus 2" OR "2019 nCoV" OR "2019nCoV" OR "2019‐novel CoV" OR "nCov 2019" OR "nCov 19" OR "coronavirus infection" OR "severe acute respiratory syndrome coronavirus 2" OR "novel coronavirus disease" OR "novel corona virus disease" OR "corona virus disease 2019" OR "coronavirus disease 2019" OR "novel coronavirus pneumonia" OR "novel corona virus pneumonia") AND (Remdesivir OR Veklury OR GS-5734 OR GS5734 OR RDV AND molnupiravir OR lagevrio OR MK-4482 OR MK4482) | 1 July 2024 |
